# Strategies for RNA Resonance Assignment by ^13^C/^15^N- and ^1^H-Detected Solid-State NMR Spectroscopy

**DOI:** 10.3389/fmolb.2021.743181

**Published:** 2021-10-20

**Authors:** Philipp Innig Aguion, Alexander Marchanka

**Affiliations:** Institute for Organic Chemistry and Centre of Biomolecular Drug Research (BMWZ), Leibniz University Hannover, Hanover, Germany

**Keywords:** RNA, solid-state NMR, assignment, resonances, MAS

## Abstract

Magic angle spinning (MAS) solid-state NMR (ssNMR) is an established tool that can be applied to non-soluble or non-crystalline biomolecules of any size or complexity. The ssNMR method advances rapidly due to technical improvements and the development of advanced isotope labeling schemes. While ssNMR has shown significant progress in structural studies of proteins, the number of RNA studies remains limited due to ssNMR methodology that is still underdeveloped. Resonance assignment is the most critical and limiting step in the structure determination protocol that defines the feasibility of NMR studies. In this review, we summarize the recent progress in RNA resonance assignment methods and approaches for secondary structure determination by ssNMR. We critically discuss advantages and limitations of conventional ^13^C- and ^15^N-detected experiments and novel ^1^H-detected methods, identify optimal regimes for RNA studies by ssNMR, and provide our view on future ssNMR studies of RNA in large RNP complexes.

## Introduction

In the last 2 decades, biomolecular solid-state NMR (ssNMR) spectroscopy has obtained a massive boost both from the progress in technical development, particularly with the advent of ultrafast magic angle spinning (MAS) probes, and from the introduction of novel isotope labeling techniques (Lu et al., 2010; Atreya, 2012; Marchanka et al., 2018a). New ssNMR studies of challenging biomolecular systems using cutting-edge technologies are being reported at an increased pace with most recent datasets having been acquired using spectrometers at the highest possible field strengths (1.2 GHz) (Callon et al., 2021; Nimerovsky et al., 2021) and under ultrafast MAS rates (111 kHz and, most recently, 150 kHz) (Penzel et al., 2019; Schledorn et al., 2020). ssNMR, in contrast to solution-state NMR, does not suffer from molecular weight (MW) limitations and therefore can be applied to various biomolecules, including membrane proteins, amyloid fibrils, and protein–protein assemblies (Castellani et al., 2002; Shi et al., 2011; Andreas et al., 2016; Quinn and Polenova, 2017). While structural ssNMR studies on proteins are well established, similar studies on free nucleic acids and nucleic acid parts of biomolecular complexes have been initiated significantly later and remain scarce due to as yet underdeveloped methodology and challenges arising from the significant spectral overlap of nucleic acid resonances (Marchanka and Carlomagno, 2014; Sreemantula and Marchanka, 2020). Nevertheless, a few impressive studies on nucleic acids have been performed in the last 2 decades. ssNMR studies of RNAs were pioneered by the Görlach group, who have not only contributed to the methodological development but also provided important insights into the structure of the ∼100 kDa (CUG)_97_ RNA repeat involved in the neuromuscular disease myotonic dystrophy (Leppert et al., 2004; Riedel et al., 2005b; a; Riedel et al., 2006). The Drobny laboratory has used ssNMR to study the structure and dynamics of 29mer HIV TAR RNA bound to an 11mer Tat peptide using site-specific ^19^F RNA labeling (Olsen et al., 2005; Huang et al., 2010; Olsen et al., 2010).

The first complete assignment of RNA resonances along with the first 3D structure of RNA in a protein–RNA complex established solely by ssNMR spectroscopy was obtained by the Carlomagno laboratory and was a major milestone in the development of ssNMR for RNAs (Marchanka et al., 2013; Marchanka et al., 2015). Our and Carlomagno’s laboratories have also been active in the characterization of protein–RNA interfaces, and we have recently determined the structure of the same protein–RNA complex by a combination of paramagnetic relaxation enhancement (PRE)-aided ssNMR and chemical shift perturbation (CSP) analysis (Ahmed et al., 2020). Finally, the first studies on RNA at 40 kHz MAS (Yang et al., 2017; Zhao et al., 2019) and MAS ≥ 100 kHz (Marchanka et al., 2018b; Aguion et al., 2021) have been performed, reporting both assignment of resonances and identification of base pairs by sensitive ^1^H-detected ssNMR spectroscopy. In the research field of phage viruses, the Goldbourt laboratory has obtained nucleotide-type assignment for very large native DNA (Sergeyev et al., 2011; Morag et al., 2014; Goldbourt, 2019) and has recently identified base pairs in native 1.2 MDa RNA from the bacteriophage MS2 (Lusky et al., 2021).

**FIGURE 1:**
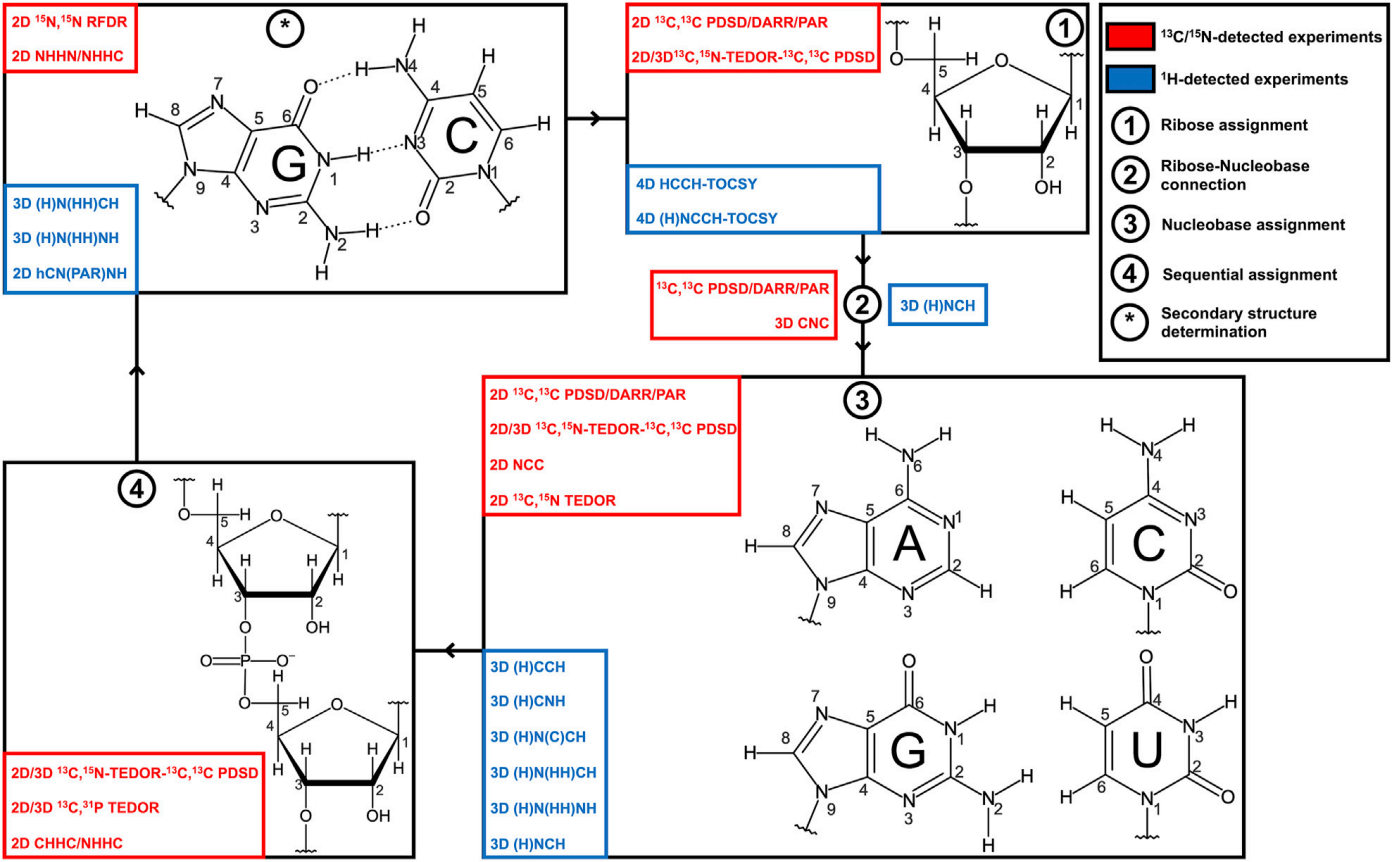
Strategies for RNA resonance assignment by ssNMR. Nucleobase and ribose structures are shown. ^13^C/^15^N-detected and ^1^H-detected experiments utilized for the different steps of the assignment protocol are shown in blue and red boxes, respectively.

Structural determination of RNA by ssNMR comprises several steps that include sample preparation, spin system–specific (assignment of resonances within a nucleotide spin system) and site-specific/sequential (assignment of defined spin systems to a specific residue within the RNA) assignment of resonances, and the collection of distance and angular restraints which are then used in structural calculations (Marchanka and Carlomagno, 2019) (Figure 1). While in some studies, unambiguous assignment of RNA resonances is not necessary to obtain valuable structural information (Olsen et al., 2005; Huang et al., 2010, 2011; Yang et al., 2017; Lusky et al., 2021), in most cases, site-specific assignment of resonances is the main limiting and crucial step in structure determination by NMR.

In this review, we describe the methods for the resonance assignment of RNA by ssNMR and compare them with solution-state NMR approaches. We provide a comprehensive description of ssNMR experiments suitable for the spin system–specific assignment of riboses and nucleobases and identification of the base-pairing pattern in RNA. Furthermore, we briefly discuss ssNMR experiments for the sequential assignment of RNA. We examine conventional ^13^C-detected and novel ^1^H-detected ssNMR methods, critically assess their strengths and limitations at different MAS frequencies, and discuss optimal MAS regimes for ssNMR studies of RNA.

### Can RNA Resonances Always Be Assigned by ssNMR?

RNAs for NMR studies are typically prepared by *in vitro* transcription (Milligan et al., 1987; Milligan and Uhlenbeck, 1989) or chemical synthesis (Beaucage and Reese, 2009). While chemical synthesis can produce RNA with sophisticated site-specific labeling, this method is limited to RNA of ∼70 nt in length and is not available in most laboratories. On the other hand, *in vitro* transcription can deliver RNA of any length labeled uniformly or selectively by nucleotide type and is potentially accessible to any laboratory. In this review, we will mostly discuss experiments suitable for the assignment of RNA that is either uniformly labeled or selectively labeled by nucleotide type.

Solution-state NMR studies of RNA have an intrinsic MW limit of ∼40 kDa (120–150 nt) and larger RNA can be assigned only partially; advanced structural studies on large RNA use many differently labeled samples and sophisticated experiments (Lu et al., 2010; Keane et al., 2015; Brown et al., 2020). While ssNMR can, in principle, be applied to RNA of any size, the feasibility of ssNMR studies is determined by the quality of sample preparation, which has a direct impact on the spectral linewidth and therefore on spectral crowding. It is commonly accepted that ^13^C linewidths smaller than 1 ppm are necessary to perform resonance assignment and obtain structural data on non–site-specific labeled samples. Due to the limited number of ssNMR RNA studies, statistics on the quality of different sample preparation techniques are very scarce; nevertheless, some patterns have been identified. Typical linewidths of lyophilized sample preparations are significantly larger than 1 ppm (Olsen et al., 2005; Huang et al., 2011) since insufficient hydration ultimately leads to large inhomogeneous broadening. As stated, linewidths greater than 1 ppm are not sufficient for the resonance assignment, which is, however, not necessary in studies utilizing site-specifically labeled RNAs (Olsen et al., 2005).

The most commonly used sample preparation method of RNA labeled uniformly or selectively by nucleotide type for both ^13^C-detected and ^1^H-detected ssNMR studies is micro (nano)-crystallization. This technique was developed for ssNMR studies of proteins (McDermott et al., 2000; Franks et al., 2005; Bertini et al., 2010) and has been successfully applied to study RNA (Huang et al., 2012; Marchanka et al., 2013; Yang et al., 2017). This method provides typical ^13^C linewidths of 1 ppm (29mer HIV TAR RNA) (Huang et al., 2012) or even 0.5 ppm for the 26mer box C/D RNA in complex with L7Ae protein (Marchanka et al., 2013; Marchanka et al., 2015). The same complex shows an ^1^H linewidth of separated C1′-H1′ resonances in the protonated ribose of 150 Hz (0.15 ppm) by ^1^H-detection at 100 kHz MAS on a 1 GHz spectrometer, while the ^1^H linewidth of imino resonances on an 850 MHz spectrometer estimates to 200–300 Hz (0.23–0.35 ppm) (Aguion et al., 2021). In the ^1^H-detected imino spectrum of 26mer DIS-HIV-1 RNA acquired at 40 kHz MAS, the linewidths of the ^1^H and ^15^N dimensions are equal to 500 Hz (0.9 ppm) and 70 Hz (1 ppm), respectively (Yang et al., 2017).

A novel ethanol precipitation method introduced by the Wang group (Zhao et al., 2019) utilizing 75% D_2_O/25% H_2_O-based buffer yields a promising ^15^N linewidth of 80 Hz (1.3 ppm) and 70 Hz (1.2 ppm) for imino resonances of 26mer DIS-HIV-1 RNA and 71mer RiboA71 domain of *add* adenine riboswitch, respectively. The ^1^H linewidth of imino resonances in these two RNAs was generously estimated to 230 Hz (0.38 ppm) and 132 Hz (0.22 ppm), respectively. While RiboA71 showed a good chemical shift dispersion so that different spin systems could be identified and even tentatively assigned based on the known solution-state chemical shifts (Zhao et al., 2019), the chemical shift range for DIS-HIV-1 was very narrow and no identification of individual spin systems was possible. It can be speculated that for the isolated 26mer DIS-HIV-1 RNA, the tertiary structure and the local order are partially lost upon EtOH precipitation, while for the well-folded riboswitch RiboA71, the tertiary structure is preserved. Further investigations into this matter are required, but this approach undoubtedly added a new technique to the repertoire of ssNMR RNA sample preparations. Unfortunately, this method cannot be applied toward protein–RNA complexes due to the instant precipitation of most proteins under such conditions.

While both microcrystallization and ethanol precipitation methods demonstrate the general feasibility of RNA resonance assignment by ssNMR, the typically obtained linewidths are significantly worse than those in solution-state NMR (< 0.1 ppm for ^1^H). It implies that nucleotide-type selective labeling is necessary to perform ^13^C-detected ssNMR studies even on short (< 30 nt) RNA (Marchanka et al., 2018a), while ^1^H-detection at MAS > 60 kHz allows us to study such RNA using a single uniformly labeled sample (Marchanka et al., 2018b). For larger RNA, site-specific or segmental labeling of short (< 30 nt) RNA stretches should be utilized to make both ^13^C-detected and ^1^H-detected ssNMR studies feasible (Marchanka et al., 2018a).

### Is There Always a Need for Complete Resonance Assignment of RNA?

Solution-state NMR spectroscopy provides rapid information about the secondary structure of RNA and identifies canonical Watson–Crick (WC) and non-WC base pairs by observation of characteristic chemical shifts of immobilized amino (NH_2_) and imino (NH) groups (Wacker et al., 2020). In solution-state NMR, site-specific assignment of ^13^C resonances is typically not necessary to site-specifically assign imino resonances and identify the RNA secondary structure. 2D ^1^H,^1^H imino NOESY/3D ^1^H,^15^N HMQC-^1^H,^1^H NOESY (Nikonowicz and Pardi, 1993) coupled with HNN-COSY (Dingley and Grzesiek, 1998; Pervushin et al., 1998) and ^1^H,^15^N-TROSY (Favier and Brutscher, 2011) experiments provide imino assignment and allow imino–imino sequential walk for the nucleotides in the base-paired and/or stacked regions. However, if complete sequential assignment and, especially, determination of the three-dimensional structure of RNA are aimed at, full assignment of RNA resonances is necessary. This task comprises several steps and includes spin system–specific assignment of all ribose atoms, determination of ribose–base connections, and assignment of base resonances, followed by sequential assignment *via* ^13^C-edited/filtered ^1^H,^1^H NOEs (Zwahlen et al., 1997; Breeze, 2000). Detailed description of solution-state NMR methods for the RNA assignment can be obtained from the classic work by the Schwalbe group (Fürtig et al., 2003) and from a few recent reviews (Scott and Hennig, 2008; Barnwal et al., 2017).

Since dipolar couplings are preserved in solid-state NMR, they can be utilized to provide rapid insights into the secondary structure of RNA by direct observation of base–base correlations. In contrast to solution-state NMR, ssNMR spectra not only show resonances from the base-paired nucleotides but also from any other immobilized nucleotides, so that observation of amino or imino resonances is not necessarily indicative of a base pairing.

**FIGURE 2:**
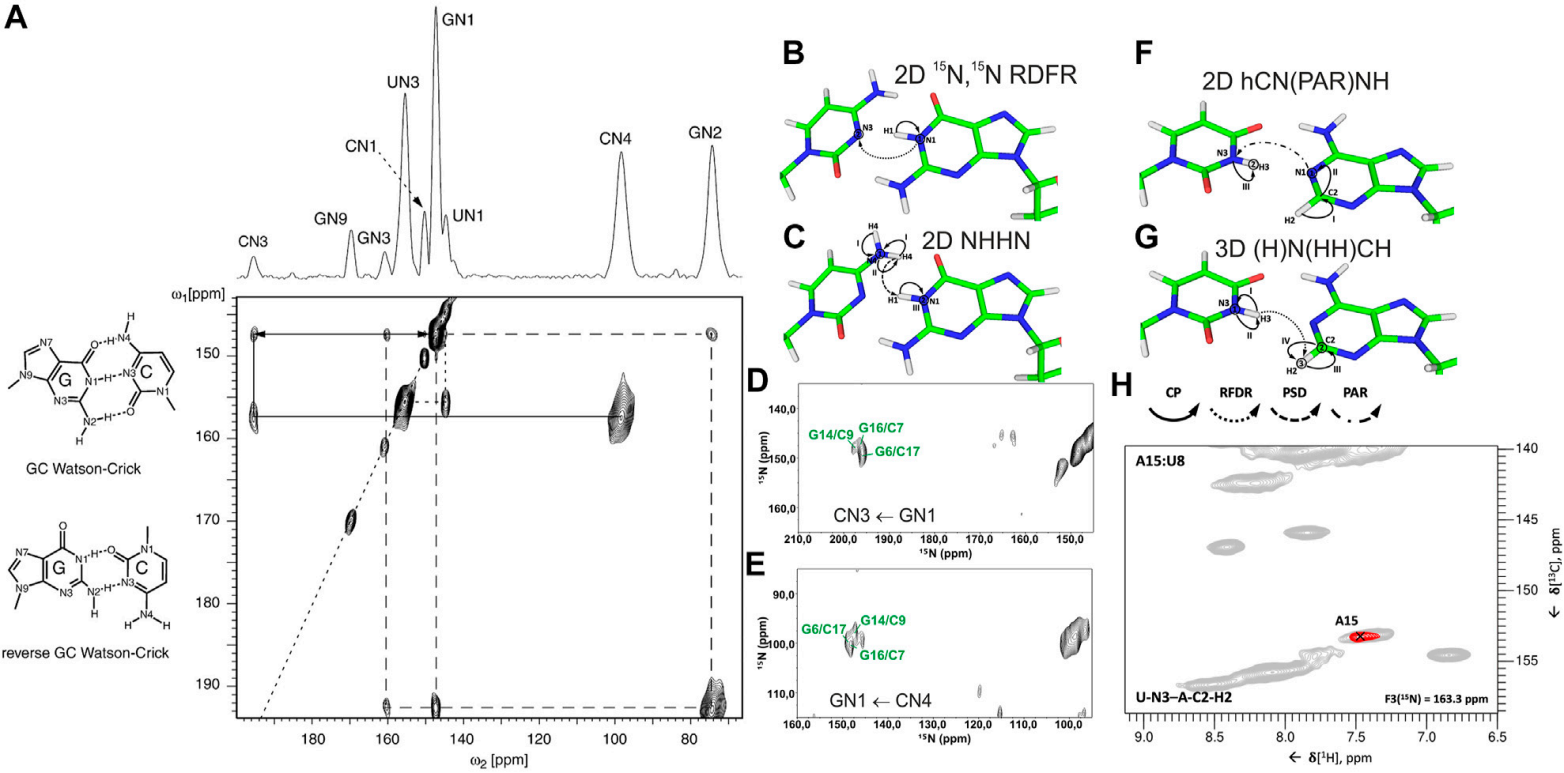
Base pair identification and spin system–specific assignment of imino nitrogens by ssNMR. **(A)** Identification of WC G:C base pair in (CUG)_97_ RNA repeat by ^15^N,^15^N RFDR experiment (Leppert et al., 2004). Magnetization transfer schemes for **(B)** ^15^N,^15^N recoupling (RFDR, PAR) experiment and **(C)** NHHN experiment shown on the example of WC G:C base pair. **(D)** 2D ^15^N,^15^N RFDR and **(E)** 2D NHHN spectra of G,C^lab^ 26mer box C/D RNA showing three assigned WC G:C base pairs (Marchanka et al., 2015). Magnetization transfer schemes for **(F)** 2D hCN(PAR)NH (Yang et al., 2017) and **(G)** 3D (H)N(HH)CH (Aguion et al., 2021) experiments **(H)** Representative 2D plane from the 3D (H)N(HH)CH spectrum shows WC base pair A15:U8 in 26mer Box C/D RNA. ^15^N frequency of N3 nitrogen in uracil is indicated. In magnetization transfer schemes, solid arrows indicate CP transfers, dotted arrows indicate homonuclear RFDR recoupling, the dashed arrow indicates PSD transfer, and the dash-dotted arrow indicates PAR transfer; Roman numbers indicate CP transfers; Arabic numbers correspond to the spectral dimensions (t_1_–t_3_). Figure **(A)** is reproduced from J. Leppert, C. R. Urbinati, S. Häfner, O. Ohlenschläger, M. S. Swanson, M. Görlach, and R. Ramachandran, Identification of NH...N hydrogen bonds by magic angle spinning solid state NMR in a double stranded RNA associated with myotonic dystrophy. Nucleic Acids Res., 2004, 32, 3, 1,177–1,183, by permission of Oxford University Press. Spectrum in **(D)** is adapted from (Marchanka et al., 2015). Spectrum in **(E)** is reprinted from (Marchanka and Carlomagno, 2019) with permission from Elsevier.

In conventional ^13^C/^15^N-detected ssNMR spectroscopy at MAS < 20 kHz, base pair information can be obtained directly by measuring ^15^N–^15^N correlations between base-paired nucleotides, either through direct dipolar transfers *via* radiofrequency-driven dipolar recoupling (RFDR) (Bennett et al., 1992) or proton-assisted recoupling (PAR) (Lewandowski et al., 2009), or *via* spin diffusion (SD)-based experiments, for example, proton-driven SD (PDSD) (Szeverenyi et al., 1982) and dipolar-assisted rotational resonance (DARR) (Takegoshi et al., 2001), or *via* proton spin diffusion (PSD) NHHN/NHHC experiments (Lange et al., 2002; Riedel et al., 2005a; Herbst et al., 2008). The Görlach laboratory has directly observed canonical WC G:C base pairs in (CUG)_97_ RNA (Figure 2A), employing both ^15^N,^15^N RFDR (Leppert et al., 2004) (Figure 2B) and NHHN (Riedel et al., 2005a) experiments (Figure 2C). In our study on 26mer box C/D RNA, we have acquired both RFDR and NHHN spectra that were sufficient for the detection of WC G:C (Figures 2D,E) and A:U base pairs. Site-specific assignment *via* imino–imino sequential walk was not possible in ^15^N-detected spectra due to poor ^15^N chemical shift dispersion and typical ^15^N linewidths ≥ 1 ppm. Recently, the Goldbourt laboratory has employed ^15^N,^15^N PDSD and ^15^N,^15^N PDSD∙RFDR experiments to identify the presence of WC G:C and wobble G:U base pairs in native 1 MDa-sized bacteriophage MS2 RNA and obtained nucleotide-type–specific assignments (Lusky et al., 2021). In all ^15^N-detected experiments described above, after the initial ^1^H-^15^N cross-polarization (CP), magnetization is evolved on ^15^N during *t*_*1*_. Following that, ^15^N magnetization is spread to nearby nitrogens, either directly by various ^15^N–^15^N recoupling schemes (Figure 2B) or indirectly through protons *via* the N→H-PSD-H→N scheme (Figure 2C). Finally, the ^15^N magnetization is recorded during *t*_*2*_.

^1^H-detected ssNMR on RNA amino and imino groups is possible in two different regimes depending on the MAS frequency used. Since imino and amino protons are exchangeable, high level of deuteration will reduce the network of ^1^H, ^1^H dipolar couplings and therefore make ^1^H resonances observable even at MAS frequencies of ∼20 kHz. The Reif and Carlomagno groups used this approach to acquire an ^1^H-^15^N dipolar-based CP-HSQC (Zhou et al., 2007) spectrum of deuterated 26mer box C/D RNA in complex with L7Ae protein at 24 kHz MAS in 90% D_2_O buffer (Asami et al., 2013). Although the recorded spectrum showed disperse imino resonances of several nucleotides, sequence-specific assignment *via* sequential walk has not been attempted due to low sensitivity. Recently, the Wang laboratory (Yang et al., 2017) has acquired proton-detected ^15^N,^15^N PAR correlations at 40 kHz MAS to obtain information about WC G:C and A:U base pairs. In their hCN(PAR)NH experiment, after initial CP transfer from ^1^H to ^13^C, a specific ^13^C-^15^N CP step is used to transfer magnetization to nitrogen atoms, whose chemical shifts are recorded in t_1_. Magnetization transfer across the base pair is achieved by ^15^N–^15^N PAR transfer with a contact time of 7 ms. A final CP transfer brings the magnetization back to ^1^H for detection (Figure 2F). Their approach was successful as they were able to confirm the presence of WC G:C and A:U base pairs in the 26mer DIS-HIV-1. Unfortunately, the low resolution in the ^1^H dimension (∼500 Hz for imino protons) did not allow the identification of different spin systems and thus the unambiguous assignment of hydrogen bonds. In our recent study at 100 kHz MAS, in view of good linewidths in the proton dimension (200–300 Hz), spin system–specific detection of base pairs was possible (Aguion et al., 2021), allowing rapid identification of almost all base pairs present in 26mer box C/D RNA. While the 2D/3D (H)N(HH)NH experiment detects WC G:C and non-WC U:U base pairs, the 2D/3D (H)N(HH)CH experiment identifies WC A:U and non-WC G:A base pairs. In the 2D/3D (H)N(HH)CH experiment (Figure 2G), after initial ^1^H-^15^N CP transfer, ^15^N chemical shifts are evolved during t_1_. After the second CP has transferred magnetization back to the protons, a ^1^H,^1^H RFDR of 0.48–0.96 ms spreads magnetization to all nearby protons within a distance of 3–4 Å. The third CP transfers magnetization to ^13^C for an optional evolution (in 3D experiment). Finally, a short read-out CP step transfers magnetization back to the protons for detection during t_2_ (t_3_ in 3D). In the 2D/3D (H)N(HH)NH experiment, the third CP step transfers magnetization to the directly attached nitrogens, where their chemical shifts evolve during t_2_ (in the optional 3D experiment). The final short ^15^N-^1^H CP read-out step transfers magnetization back to the protons for detection during t_2_ (t_3_ in 3D). Despite efficient identification of both WC and non-WC base pairs (Figure 2H), sequential imino–imino walk as performed in solution-state NMR was not feasible due to 1) low sensitivity and 2) strong signal overlap.

Presented case studies show that ssNMR allows rapid identification of the type and number of base pairs present in the RNA. However, sequence-specific assignment of base pairs and therefore RNA secondary structure determination based on amino and imino fingerprints alone are not possible and, hence, complete assignment of RNA resonances is necessary. The above will require their correlation with the well-resolved nucleobase carbons C6/C8 (C6-H6/C8-H8 groups) and then with the ribose C1′ (C1′-H1′ groups). Finally, nucleotides should be connected sequentially through ^1^H–^1^H and/or ^13^C–^13^C correlations (Figure 1).

In the following sections, we will discuss in detail experimental strategies for the resonance assignment of different RNA moieties by ssNMR.

### Ribose Assignment

**FIGURE 3:**
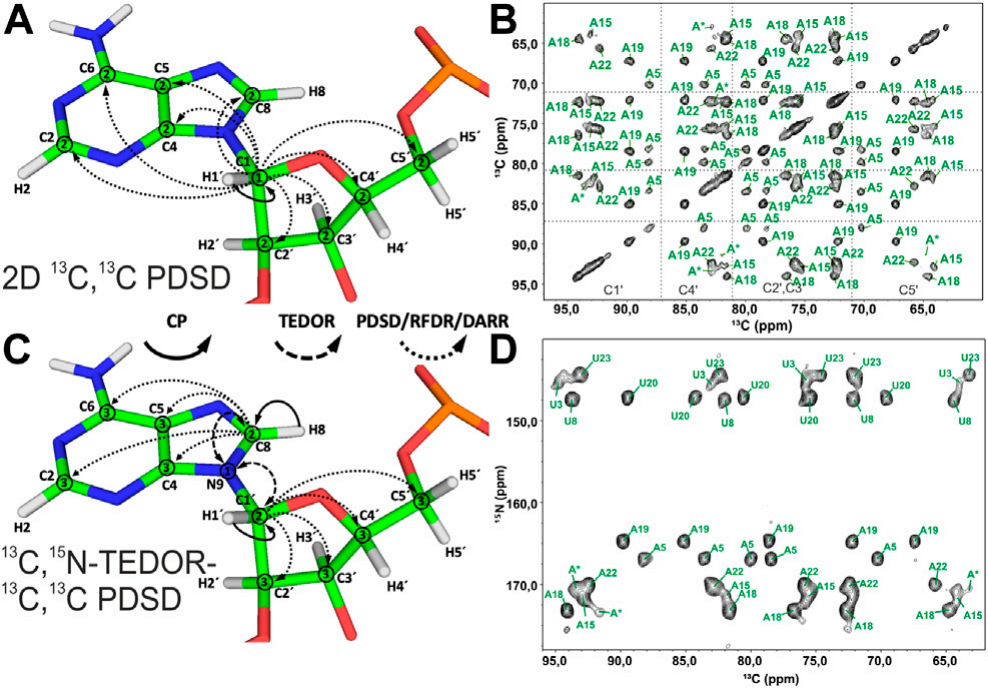
Ribose assignment by ^13^C-detected ssNMR. **(A)** Magnetization transfer in homonuclear ^13^C,^13^C recoupling experiment (PDSD, DARR, and PAR) for the spin system–specific assignment of carbon resonances in nucleotides shown on the example of an adenosine. **(B)** Zoom in on the ribose region of ^13^C,^13^C PDSD spectra of the A^lab^-26mer box C/D RNA measured at the mixing time of 100 ms. **(C)** Magnetization transfer in heteronuclear ^13^C,^15^N-TEDOR-^13^C,^13^C PDSD experiment shown on the example of an adenosine. **(D)** Ribose region of the ^13^C,^15^N-TEDOR-^13^C,^13^C PDSD spectrum of the A,U^lab^-26mer box C/D RNA measured at TEDOR and PDSD mixing times of 3.2 and 100 ms, respectively. In magnetization transfer schemes, solid arrows indicate CP transfers, dashed arrows indicated TEDOR transfer, and dotted arrows indicate homonuclear ^13^C,^13^C recoupling; Arabic numbers correspond to the spectral dimensions (t_1_–t_3_). The spectrum in **(B)** is adapted with permission from (Marchanka et al., 2013), © John Wiley and Sons, 2013. The spectrum in **(D)** is adapted from (Marchanka et al., 2015).

Spin system–specific assignment of ribose resonances by conventional ^13^C-detected ssNMR at MAS frequencies < 20 kHz can be achieved using a multitude of correlation schemes provided a satisfactory spectral linewidth is obtained. In the pioneering study by the Görlach group, mostly intra-ribose and partially ribose–base correlations were obtained using symmetry-based adiabatic ZQ recoupling experiments (Riedel et al., 2004). However, due to the low chemical shift resolution arising from limitations in sample preparation, spin system–specific assignment was not possible despite only three different nucleotides being present in the (CUG)_97_ RNA. The Drobny group (Huang et al., 2012) acquired ^13^C,^13^C PDSD (Szeverenyi et al., 1982) experiments on the selectively uracil-labeled TAR RNA. Also, there, very narrow carbon chemical shift dispersion together with poor resolution impeded any spin system–specific resonance assignments. In our study on 26mer box C/D RNA, we have exploited 2D ^13^C,^13^C PDSD (Figure 3A) to correlate intra-ribose resonances and even to obtain ribose–base correlations (Marchanka et al., 2013). While 100 ms PDSD mixing was sufficient to obtain a full set of intra-ribose correlations (Figure 3B), 500 ms mixing additionally provided not only intra-base correlations but also an almost complete set of ribose–base and several inter-nucleotide correlations (s. below). Despite good ^13^C linewidths of 0.5 ppm and usage of nucleotide-type selective labeling (Marchanka et al., 2013; Marchanka et al., 2018a), homonuclear ^13^C,^13^C spectra yielded the assignment of less than half of the nucleotides of 26mer box C/D RNA. The assignment process is hampered particularly by significant spectral crowding of C2′/C3′ carbons, and furthermore, the lack of a proton dimension does not help to lift the ambiguity.

A MAS regime of 20 kHz < ω_R_ < 60 kHz is less suitable for the ribose assignment in RNA labeled uniformly or selectively by nucleotide type. First, since ribose protons are not exchangeable, acquisition of NMR spectra in deuterated buffer does not bear any advantage. Coherence lifetimes of ribose protons are unfavorable at MAS frequencies < 60 kHz (Marchanka et al., 2018b), so that ^13^C-detected ssNMR must be applied. While 3.2 mm probes (maximum MAS frequency = 24 kHz) have optimal ^13^C sensitivity, the smaller rotor size in 2.5 and 1.7 mm probes attenuates ^13^C sensitivity due to a smaller sample volume. Second, pure SD-based experiments (PDSD and DARR) cannot work well at MAS > 20 kHz; therefore, advanced recoupling schemes must be utilized to correlate ribose resonances in this regime. These mixing schemes may include finite-pulse (fp) RFDR (Bennett et al., 1992; Nishiyama et al., 2014), R-symmetry–driven SD (Hou et al., 2011), mixed rotational and rotary resonance (MIRROR) (Scholz et al., 2008), phase-alternated recoupling irradiation (PARIS) (Weingarth et al., 2009), combined R2-symmetry–driven sequences (CORD) (Hou et al., 2013), and many others. A recent review by the Hou group provides a comprehensive description of different recoupling techniques at fast MAS (Ji et al., 2021). Many first-order homonuclear dipolar recoupling sequences (e.g., fpRFDR) suffer from dipolar truncation (Bayro et al., 2009), which may impair observation of long-range correlations, whereas second-order sequences (SD-based, RF-assisted SD, and PAR) are free from this effect (Ji et al., 2021). Regardless of the chosen type of homonuclear recoupling, all ^13^C,^13^C correlation experiments have a similar form. After an initial short CP transfer from ^1^H to ^13^C, magnetization is evolved on the starting ^13^C during t_1_. Subsequently, carbons are connected by one of the recoupling schemes. Finally, ^13^C chemical shifts are recorded during t_2_ (Figure 3A).

Since N1 and N9 nitrogens in pyrimidines and purines, respectively, have very distinct chemical shifts, the ^15^N dimension can be added to improve assignment by separation of ribose resonances of different nucleotide types. Optimal separation can be achieved by acquisition of, for example, ^13^C,^15^N TEDOR (Jaroniec et al., 2002) or ^13^C,^15^N TEDOR-^13^C,^13^C PDSD (Riedel et al., 2005b; Daviso et al., 2013) experiments. In the TEDOR-PDSD experiment, the ^13^C magnetization (C1′) is prepared *via* a short ^1^H,^13^C CP step. During a short ^13^C,^15^N TEDOR transfer, the magnetization is propagated to nearby nitrogens (N1 and N9 in pyrimidines and purines, respectively). After t_1_ evolution on ^15^N, the magnetization is transferred back to ^13^C. A subsequent ^13^C,^13^C PDSD or DARR step spreads the magnetization into ^13^C spins of ribose (C2′-C5′), whose chemical shifts are recorded during t_2_ (t_3_ in 3D) (Figure 3C). An optional ^13^C evolution step before PDSD (DARR) yields the 3D TEDOR-PDSD experiment which improves resolution at the cost of sensitivity. In addition to improving ribose assignment (Figure 3D), TEDOR-PDSD provides ribose–base connections and improves the assignment of nucleobase carbons (*vide infra*).

**FIGURE 4:**
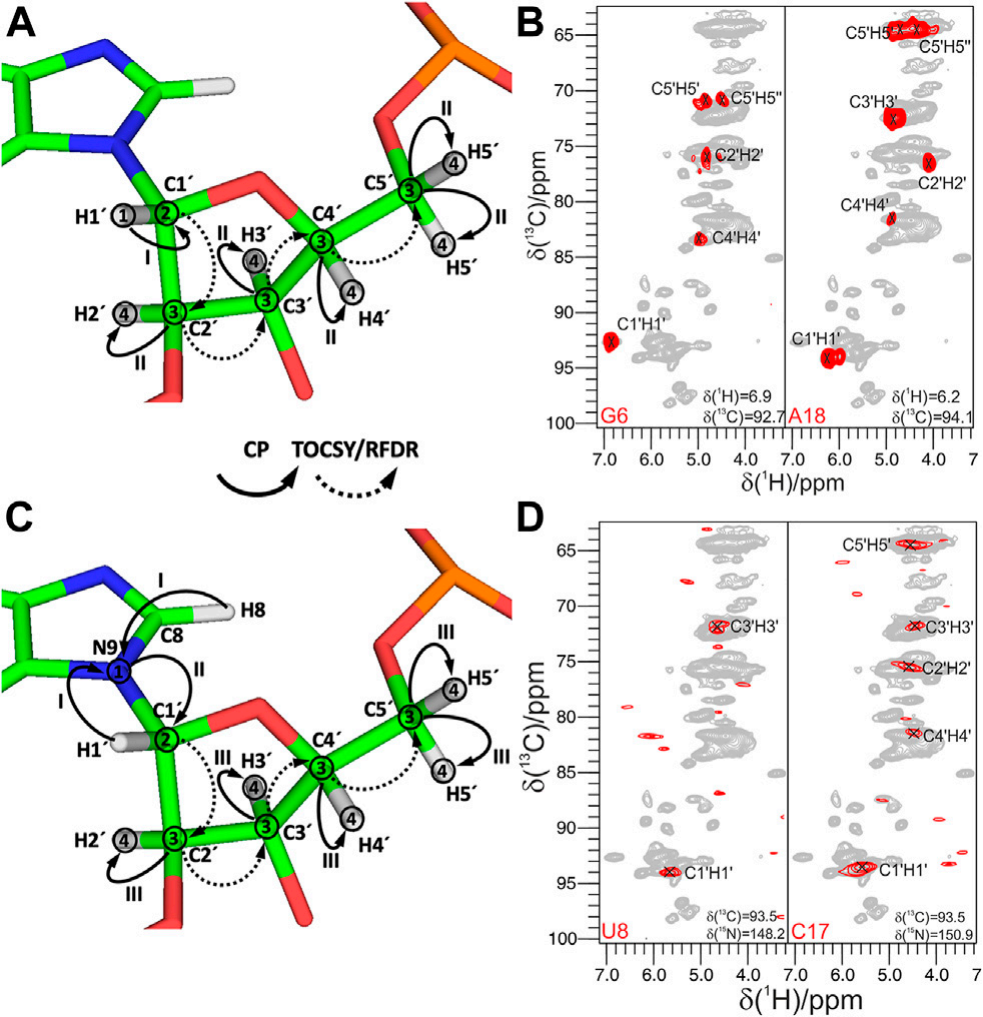
Ribose assignment by ^1^H-detected ssNMR. **(A,C)** Magnetization transfer for **(A)** 4D HCCH-TOCSY and **(C)** 4D (H)NCCH-TOCSY experiments. Solid arrows indicate CP transfers and dotted arrows indicate homonuclear ^13^C,^13^C TOCSY/RFDR recoupling; Arabic numbers indicate spectral dimensions (t_1_–t_4_); Roman numbers indicate CP transfers. Assignment of ribose spin systems with **(B)** 4D HCCH-TOCSY and **(D)** 4D (H)NCCH-TOCSY experiments. In **(B)** representative 2D planes from the 4D experiment show the spin systems of G6 and A18. ^1^H and ^13^C frequencies are indicated in each panel. In **(D)** representative 2D planes from the 4D experiment show the spin systems of U8 and C17. ^13^C and ^15^N frequencies are indicated in each panel. For reference, the red contours of either HCCH-TOCSY or 4D (H)NCCH-TOCSY spectra are overlaid onto 2D CP-HSQC spectra (in gray). The HCCH-TOCSY spectrum was recorded on an 800 MHz spectrometer at a MAS frequency of 100 kHz, while the (H)NCCH-TOCSY spectrum was recorded on a 1 GHz spectrometer and at 100 kHz MAS. Figures **(B)** and **(D)** are reproduced from (Marchanka et al., 2018b) with permission from the Royal Society of Chemistry.

^1^H-detected ssNMR at MAS frequencies > 60 kHz and in particularly ≥ 100 kHz opens new avenues for structural characterization of biomolecules, significantly improving resolution and increasing the sensitivity per unit of the sample. Such probeheads operate with significantly smaller rotors that ultimately reduce the sample volume/number of spins packed into the ssNMR rotor and therefore attenuate sensitivity. Due to the optimized coil sensitivity, increased fill factor, and narrowed lines, overall sensitivity is not reduced as a cube of the rotor diameter (Schledorn et al., 2020); nevertheless, exclusively ^1^H detection with the sensitivity increased by a factor of ${\left(\gamma_{H}/\gamma_{C}\right)}^{3/2}=8$ can compensate for the smaller rotor size. In our experience, a MAS regime where ω_R_ ≥ 100 kHz is optimal for RNA studies by ^1^H-detected ssNMR. Coherence lifetimes of both H1′ and H2′-H5′ protons increase significantly at MAS frequencies above 60 kHz and reach 4.2 and 1.7 ms, respectively, at 109 kHz MAS (Marchanka et al., 2018b). In addition to many dipolar mixing schemes that can be used at MAS ≥ 100 kHz, scalar ^13^C couplings can be utilized to correlate all carbons in the ribose with each other in a manner typically utilized in solution-state NMR (Hu et al., 1998). J-coupling–based correlation spectroscopy becomes possible due to the long ^13^C T_1ρ_ relaxation times of 50 ms at ≥ 100 kHz MAS (Marchanka et al., 2018b). In our study, we have implemented a 4D HCCH-TOCSY experiment utilizing low-power 20 kHz WALTZ-16 (Shaka et al., 1983) mixing of a length of 25 ms, which allows us to fully correlate all CH groups in the ribose ring (Figures 4A,B). In the 4D HCCH-TOCSY, after t_1_ evolution on the starting proton, magnetization is transferred by a short CP to directly attached ^13^C. After t_2_ evolution on this starting carbon, magnetization is transferred to all carbons in the ribose by WALTZ-16 mixing. After evolution during t_3_ on the final carbon, magnetization is transferred by a short CP to ^1^H for detection during t_4_ (Figure 4A). This experiment has been acquired utilizing non-uniform sampling (NUS) (Paramasivam et al., 2012; Sergeyev et al., 2017) and required 68 h of measurement time. To explore the feasibility of dipolar coupling–based transfer in the ribose at 100 kHz MAS, we have acquired ^13^C,^13^C fpRFDR spectra with 8 and 16 ms of mixing time. A full set of intra-ribose correlations has been obtained from both 3D (H)CCH spectra acquired with high sensitivity using uniform sampling within 40 h.

Despite good resolution in ^1^H-detected 4D HCCH-TOCSY or 3D (H)CCH-fpRFDR spectra, three nucleotides in the helical regions were not assigned due to low dispersion of the C1′ and H1′ chemical shifts (Marchanka et al., 2018b). Similar to ^13^C-detected experiments, the N1/N9 dimension can be introduced to resolve spectral crowding at the price of sensitivity. In the 4D (H)NCCH-TOCSY experiment (Figure 4C), after a long ^1^H-^15^N CP transfer, magnetization is evolved on N1 and N9 during t_1_. Subsequently, magnetization is transferred by a specific ^13^C-^15^N CP step to the C1′ carbon in the ribose. From here on, the magnetization path follows one of the HCCH-TOCSY experiments and delivers a set of well-resolved N1/N9-C1′-CX′-HX′ correlations after 100 h of measurement time, albeit with low sensitivity (Figure 4D).

### Ribose–Nucleobase Connection

**FIGURE 5:**
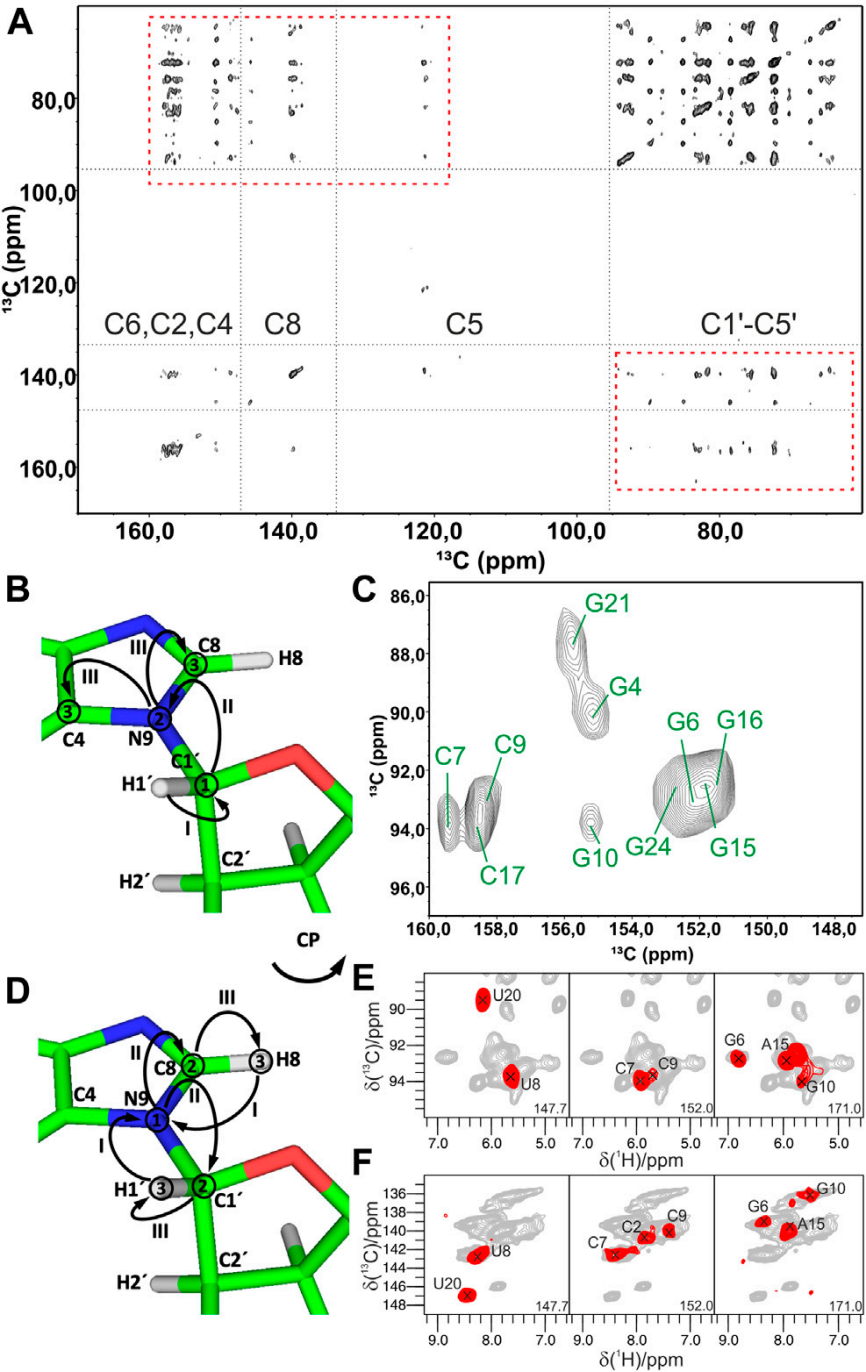
Experiments for the ribose–nucleobase correlations. **(A)** ^13^C,^13^C PDSD spectrum of the A^lab^-26mer box C/D RNA measured at 500 ms PDSD mixing time. Ribose–base cross-peaks are highlighted by rectangles. **(B,C)** ^13^C-detected 3D CNC experiment. **(B)** Magnetization transfer in CNC experiment shown on the example of a purine; **(C)** 2D C_ribose_–C_base_ projection of the 3D CNC spectrum of G,C^lab^ 26mer box C/D RNA. Spectra in figures (A,C) were acquired on a 700 MHz spectrometer at 13 kHz MAS. **(D–F)** ^1^H-detected 3D (H)NCH experiment. **(D)** Magnetization transfer shown on the example of a purine. **(E,F)** Representative ^13^C–^1^H cross-sections from the (H)NCH spectra show either ribose N1/N9–C1′–H1′ **(E)** or base N1–C6–H6/N9–C8–H8 **(F)** correlations. ^15^N frequencies are indicated in each panel. (H)NCH spectra were recorded on a 1 GHz spectrometer at 111 kHz MAS. In magnetization transfer schemes, solid arrows indicate CP transfers; Arabic numbers correspond to the spectral dimensions (t_1_–t_3_); Roman numbers indicate CP transfers. Figures **(A,C)** are adapted with permission from (Marchanka et al., 2013), © John Wiley and Sons, 2013. Figures **(E,F)** are reproduced from (Marchanka et al., 2018b) with permission from the Royal Society of Chemistry.

The next step of the resonance assignment protocol is the connection of riboses to the nucleobases, which can, in principle, be obtained in ssNMR by long-range carbon–carbon correlations utilizing previously discussed homonuclear recoupling schemes, for example, PDSD (Szeverenyi et al., 1982), DARR (Takegoshi et al., 2001), RFDR/fpRFDR (Bennett et al., 1992), PAR (De Paëpe et al., 2008), and others (Ji et al., 2021) (Figure 5A). Due to the two-bond distance between ribose C1′ and nucleobase C2/C6 and C4/C8 carbons, dipolar truncation (Bayro et al., 2009) can impair the efficiency of first-order recoupling schemes, so second-order dipolar recoupling (e.g., PDSD, DARR, PAR, and CORD) should be preferred depending on the MAS frequency. While in our studies, we have used the PDSD scheme at MAS < 20 kHz (Marchanka et al., 2013) and have employed fpRFDR at 100 kHz, various recoupling schemes (Ji et al., 2021) can be utilized for this purpose. Their efficacies toward RNA have to be evaluated in future studies.

As discussed previously in the ribose assignment section, spectral resolution in ^13^C (and ^1^H) dimensions might not be enough for the spin system assignment using homonuclear recoupling schemes. As for ribose assignment experiments, acquisition of ^15^N-edited spectra resolves nucleotides by their N1/N9 chemical shifts and facilitates unambiguous ribose–base correlation.

In ^13^C-detected ssNMR spectroscopy, ribose and base resonances can be connected by the CNC-type experiment (Franks et al., 2005; Schuetz et al., 2010) (Figure 5B), which is similar to the HCNCH (Sklenar et al., 1993) experiment in liquids. After initial CP from H1′ to C1′, magnetization is evolved on C1′ during t_1_. Next, a long SPECIFIC-CP step (Baldus et al., 1998) transfers magnetization to N1 and N9 in pyrimidines and purines, respectively, where it evolves during t_2_. Finally, during a last SPECIFIC-CP step, magnetization is transferred to C2/C6 and C4/C8 in pyrimidines and purines, respectively, for the detection during t_3_ (Marchanka et al., 2013). ^13^C detection at MAS < 20 kHz allows straightforward acquisition of ^13^C signals of both protonated and non-protonated carbons and provides well-separated C1′-N1-C2/C4 and C1′-N9-C4/C8 chemical shifts for pyrimidines and purines, respectively (Figure 5C). Due to the very distinct chemical shifts of N1 and N9 nitrogens, just two differently double nucleotide-type selectively labeled samples are sufficient to obtain such correlations for all nucleotides in RNA. This experiment can be acquired in the ribose-to-base or base-to-ribose direction. In our experience, ribose-to-base transfer was more efficient. Moreover, C1′ → N1/N9 → C2/C4 correlations were more intense than C1′ → N1/N9 → C6/C8 due to a shorter coherence lifetime caused by the increased dipolar relaxation in the latter case. Due to the two low-γ specific CP transfers and the intrinsically low sensitivity of ^13^C detection, a single good-quality 3D CNC spectrum required more than 120 h of experimental time on a 700 MHz Bruker spectrometer. However, this experiment provides assignment for C4 and C2 carbons in purines and pyrimidines, respectively, whose assigned chemical shifts are very rarely reported in the BMRB database (Ulrich et al., 2007) (s. below). The same experiment can be acquired at a MAS regime of 20 kHz < ω_R_ < 60 kHz; however, its sensitivity will be compromised due to the necessity of ^13^C-detection at decreased sample volume (see previous section).

Ribose–nucleobase correlations can be obtained by ^1^H-detected experiments at MAS > 60 kHz on a single uniformly labeled ^13^C,^15^N RNA sample (Marchanka et al., 2018b). In the ^1^H-detected (H)NCH experiment, after long ^1^H-^15^N CP transfer and evolution on ^15^N during t_1_, either ribose- or base-tuned band-selective CP transfers the magnetization either to C1′ or to C6/C8 for the evolution during t_2_. A final short CP transfers magnetization to directly bound H1′ or H6/H8 protons for the detection during t_3_ (Figure 5D). This experiment provides a set of either ribose-specific N1/N9–C1′–H1′ or base-specific N1–C6–H6/N9–C8–H8 correlations in pyrimidines/purines, respectively (Figures 5E,F). Due to the high sensitivity of ^1^H detection, good quality ribose- and base-specific spectra have been obtained in 12 and 19 h, respectively (Marchanka et al., 2018b). While the (H)NCH experiment provides unambiguous correlation of ribose and base CH groups with their corresponding N1/N9 nitrogens, ribose–base correlations obtained by these two experiments might be ambiguous, since they share only N1/N9 chemical shifts. The narrow chemical shift range of N1/N9 nitrogens is exacerbated by intrinsically worse linewidths in ssNMR compared to solution-state. To lift the ambiguity, an additional ^1^H evolution period can be added before the first ^1^H-^15^N CP step, yielding the 4D HNCH experiment. In such a spectrum, chemical shifts in the indirect proton dimension (H6 or H8) and N1/N9 represent the nucleobase, while N1/N9, C1′ and H1′ chemical shifts correspond to the ribose.

### Nucleobase Assignment

After resonances of riboses are assigned and correlated with the nucleobase C6-H6/C8-H8 groups, assignment of resonances in the nucleobase is performed. This is not a trivial task, particularly in ^1^H-detected NMR spectroscopy, due to the low density of protons in RNA nucleobases. The majority of reported assigned carbons and nitrogen RNA chemical shifts in the BMRB database belong to either protonated nuclei or nuclei directly attached to the protonated carbons. The fraction of assigned chemical shifts for C4 and C5 carbons in purines is very low and estimates to only 1.8% (72/3,921) and 2.0% (79/3,921), respectively, of all assigned base carbon chemical shifts in the BMRB database. Sensitive dipolar coupling–based transfer in ssNMR provides an unprecedented opportunity for the assignment of these otherwise not-easy-to-access carbons.

C2/C6 and C4/C8 carbons in pyrimidines and purines, respectively, are assigned by the 3D CNC experiment acquired at MAS < 20 kHz, as described in the section above. H6-C6 and H8-C8 groups are assigned by the (H)NCH experiment at ultrafast MAS.

Strategies for the assignment of the remaining carbons and nitrogens in the nucleobase depend strongly on if either ^13^C- or ^1^H-detection is utilized.

**FIGURE 6:**
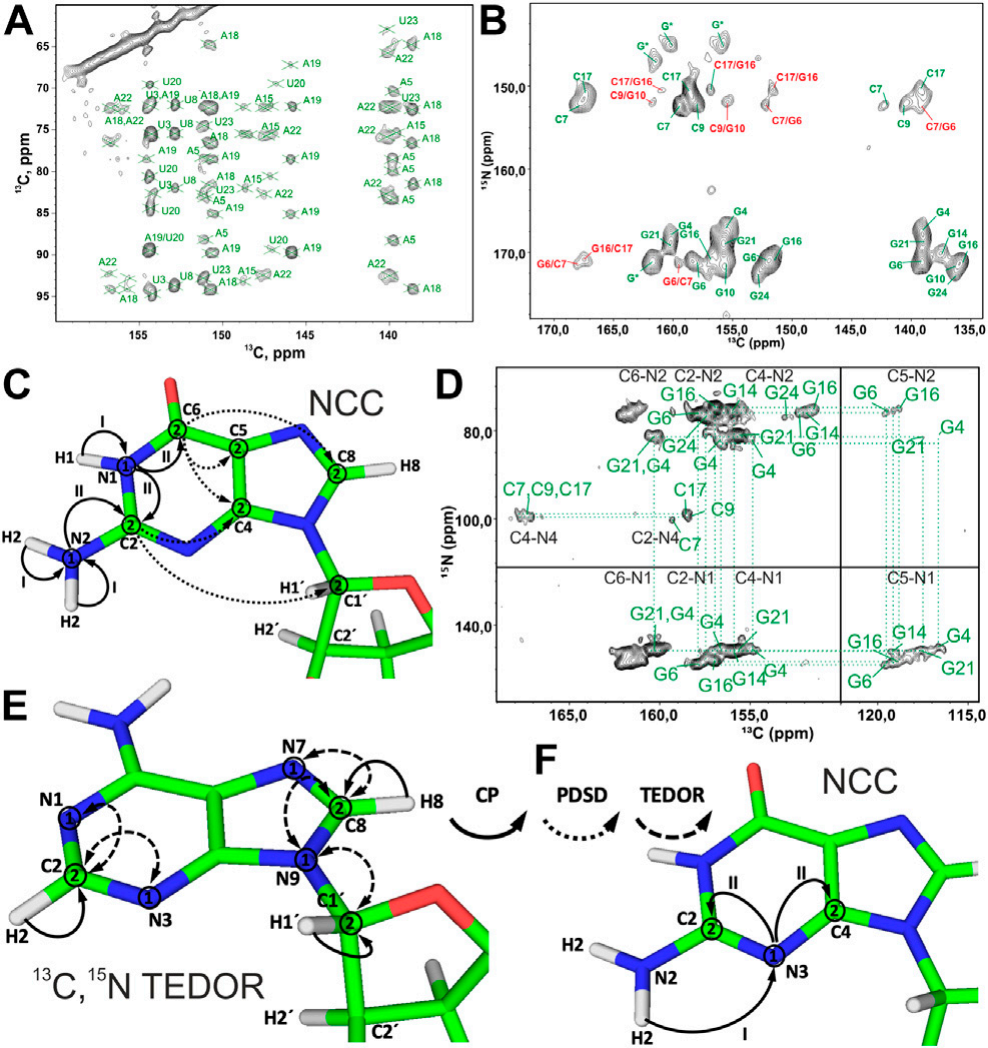
Nucleobase assignment by ^13^C-detected ssNMR. **(A)** Zoom in on the ribose–base region of the ^13^C,^13^C PAR spectrum of the A,U^lab^-26mer box C/D RNA acquired at 10 ms PAR mixing time. **(B)** Zoom in on the base region of the ^13^C,^15^N-TEDOR-^13^C,^13^C PDSD spectrum of the G,C^lab^ 26mer box C/D RNA. Intra- and inter-nucleotide correlations are labeled in green and red, respectively. **(C,D)** NCC experiment for the assignment of nitrogen and carbon resonances in the nucleobases. **(C)** Magnetization transfer in NCC experiment shown on the example of a guanosine. **(D)** 2D NCC spectrum of G,C^lab^ 26mer box C/D RNA acquired at 150 ms PDSD mixing time. Magnetization transfer in **(E)** ^13^C,^15^N TEDOR experiment for the assignment of N7 nitrogens in purines and N1/N3 nitrogens in adenosines and **(F)** in the modified NCC experiment for the assignment of N3 nitrogens in cytidines and guanosines. In magnetization transfer schemes, solid arrows indicate CP transfers, dotted arrows indicate PDSD transfers, and dashed arrows indicate TEDOR transfers; Arabic numbers correspond to spectral dimensions (t_1_–t_2_); Roman numbers indicate CP transfers. Spectra in figures **(A,B)** and **(D)** were acquired on a 700 MHz spectrometer at 16 kHz MAS and 13 kHz MAS, respectively. The spectrum in **(B)** is adapted from (Marchanka et al., 2015). The spectrum in **(D)** is adapted with permission from (Marchanka et al., 2013) © John Wiley and Sons, 2013.

At MAS frequencies < 20 kHz, using ^13^C-detection, ^13^C,^13^C recoupling schemes discussed above for the ribose assignment can be used to obtain carbon assignment in the nucleobase. 500 ms PDSD recoupling (Figure 5A) or 8–16 ms PAR (Figure 6A) were found to be sufficient to obtain many, although overlapping, correlations in the nucleobases of RNA selectively labeled by nucleotide type. As for ribose assignment, inclusion of the nitrogen dimension can reduce spectral crowding. The previously described ^13^C,^15^N TEDOR-^13^C,^13^C PDSD experiment (Riedel et al., 2005b; Daviso et al., 2013) can provide assignment of carbon resonances in nucleobases by connecting their chemical shifts to the chemical shifts of assigned carbons in the nucleobase (C6/C8) and the ribose (C1′) (Figure 6B).

The NCC experiment (Pauli et al., 2001; Igumenova et al., 2004; Franks et al., 2005) is employed to connect amino and imino nitrogens with nucleobase and (partially) ribose carbons (Marchanka et al., 2013). After an initial short CP from ^1^H to ^15^N, ^15^N chemical shifts evolve during t_1_ and yield the frequencies of N6 (A), N4 (C), N1, N2 (G), and N3 (U). Following this, the magnetization is transferred by SPECIFIC-CP to the nearby carbons, N6→C6 (A), N4→C4 (C), N1→C2/C6 and N2→C2 (G), and N3→C2/C4 (U). Here, magnetization can be optionally evolved during t_2_ or spread directly by PDSD to all base carbons and C1′ for the detection during t_2_ or t_3_ (in the 3D version) (Figures 6C,D). Needless to say, diverse ^13^C,^13^C recoupling schemes can be utilized instead of PDSD. The NCC experiment can, in theory, provide unambiguous assignment for all protonated nitrogens and all carbons in the nucleobase if protonated nitrogens can be uniquely correlated with well-resolved carbons in the nucleobase (C2/C6, C4/C8) or C1′. However, the poor chemical shift dispersion of ^15^N amino and imino resonances usually renders assignment inconclusive, especially in helical regions.

Assignment of the remaining non-protonated nitrogens in the base is important for characterization of non-WC base pairs, especially G:A and U:U. Assignment of N7 nitrogen in adenosines and guanosines can be readily obtained from the ^13^C,^15^N TEDOR (Jaroniec et al., 2002) experiment that provides (H8)C8-N7 correlations. Assignment of N1 and N3 nitrogens in adenosines is obtained from the same-type TEDOR experiment by acquisition of (H2)C2-N1/N3 correlations (Figure 6E). To obtain site-specific assignment of these N1/N3 nitrogens, C2 carbons have to be correlated with C6/C8 carbons by any of the ^13^C,^13^C correlation schemes discussed above.

The assignment of non-protonated nitrogens (N3) in both cytidines and guanosines is the most challenging task, due to the absence of any protons in their close vicinity. It could, in principle, be obtained from a modified NCC experiment, where a long initial ^1^H-^15^N CP transfers the magnetization from the distant (r = 2.4 Å) amino protons H41/H42 (C) or H21/H22 (G) to the N3 nitrogen. ^15^N chemical shifts evolve during t_1_, and then magnetization is transferred to the C2 and C4 carbons, where it is recorded during t_2_ (Figure 6F). It is uncertain if 1) T_1ρ_ during the H41/H42→N3 and H21/H22→N3 transfer is long enough to allow for the efficient long-range magnetization transfer and 2) resolution of ^15^N and ^13^C resonances are good enough to allow for the spin system–specific assignment. However, N3 of cytidine can be easily assigned indirectly through base-paired guanosine in the WC G:C base pair. Here, the cross-strand N–N distance of ∼2.0 Å allows the straightforward connection of N3 in cytidine with N1 in guanosine using, for example, ^15^N,^15^N RFDR or ^15^N,^15^N PAR correlations (Figure 2B).

While ^13^C-detected ssNMR allows almost complete assignment of carbons and nitrogens in the nucleobases using only two different types of experiments, namely, CNC and NCC, ^1^H-detected ssNMR on RNA nucleobases is more challenging. Despite the significant advantages of 1) additional spectral dimensions and 2) higher sensitivity, ^1^H detection obviously requires that the magnetization transfer pathway ends on a proton. As is known from solution-state NMR, many different types of experiments are necessary to assign RNA nucleobases (Fürtig et al., 2003).

Very recently, we have addressed this challenge and have published the set of ssNMR experiments that allow assignment of nucleobase resonances (Aguion et al., 2021).

**FIGURE 7:**
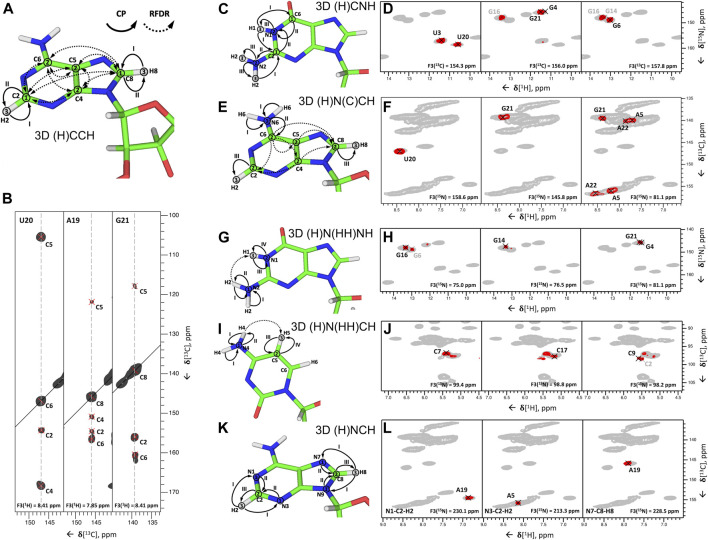
Nucleobase assignment by ^1^H-detected ssNMR. **(A,C,E,G,I,K)** Magnetization transfers for **(A)** 3D (H)CCH, **(C)** 3D (H)CNH, **(E)** 3D (H)N(C)CH, **(G)** 3D (H)N(HH)NH, **(I)** 3D (H)N(HH)CH experiments, and **(K)** 3D(H)NCH experiments, shown on the example of adenosine **(A,E,K)**, guanosine **(C,G),** and cytidine **(I)**. Solid arrows indicate CP transfers and dotted arrows indicated RFDR transfers; Arabic numbers indicate spectral dimensions (t_1_–t_3_); Roman numbers indicate CP transfers. **(B,D,F,H,J,L)** Assignment of base spin systems in the 26mer Box C/D RNA with **(B)** 3D (H)CCH, **(D)** 3D (H)CNH, **(F)** 3D (H)N(C)CH, **(H)** 3D (H)N(HH)NH, **(J)** 3D (H)N(HH)CH, and **(L)** (H)NCH experiments. In **(B)** representative 2D ^13^C–^13^C planes extracted from the 3D (H)CCH spectrum show correlations of all intra-nucleotide nucleobase carbons for the nucleotides U20, A19, and G21. In **(D)** representative 2D planes extracted from the 3D (H)CNH experiment show correlations of imino nitrogens with distinct nucleobase carbon resonances for the nucleotides U3, U20, G4, G16, G21, G6, and G14. In **(F)** representative 2D planes extracted from the 3D (H)N(C)CH experiment show correlations of imino and amino nitrogens with protonated nucleobase carbons for the nucleotides U20, G21, A5, and A22. In **(H)** representative 2D planes extracted from the 3D (H)N(HH)NH experiment show correlations of amino and imino nitrogens of guanosines for the nucleotides G16, G6, G14, G21, and G4. In **(J)** representative 2D planes extracted from the 3D (H)N(HH)CH experiment show correlations of amino nitrogens with protonated carbons of cytidines for the nucleotides C7, C17, C9, and C2. In **(L)** representative 2D planes extracted from the 3D (H)NCH experiment show correlations of non-protonated nitrogens with protonated carbons for the nucleotides A19 and A5. ^13^C or ^15^N frequencies are indicated in each panel. Peaks labeled in gray indicate that the peak maximum is not in the plane shown here. For reference in panels **(D,F,H,J,L)**, the red contours of 3D H(C)NH and (H)N(HH)NH spectra **(D,H)** are overlaid onto 2D ^1^H-^15^N CP-HSQC spectra (in gray), while red contours of 3D (H)N(C)CH, (H)N(HH)CH and (H)NCH spectra are overlaid onto 2D ^1^H-^13^C CP-HSQC spectra (in gray), tailored either for the C2-H2/C6-H6/C8-H8 **(F,L)** or the ribose/C5-H5 spectral regions **(J)**. All spectra were recorded on an 850 MHz spectrometer at a MAS frequency of 100 kHz (Aguion et al., 2021). All spectra are adapted from (Aguion et al., 2021).

In the first step, all nucleobase carbons are correlated with the previously assigned C6-H6 (pyrimidines) or C8-H8 (purines) groups using ^13^C,^13^C fpRFDR recoupling. This step is accomplished by the 3D (H)CCH experiment, which starts with a long ^1^H-^13^C CP step to gain sufficient ^13^C magnetization on both protonated and non-protonated carbons. After t_1_ evolution on ^13^C, a phase-cycled selective inversion pulse cancels the signals of the ribose ring. Following this, 8 ms-long ^13^C,^13^C fpRFDR recoupling transfers magnetization to nearby carbons, whose chemical shifts are recorded during t_2_. Finally, the ^13^C magnetization is transferred by a short read-out CP to directly attached protons for the detection during t_3_ (Figure 7A). This experiment correlates all nucleobase carbons with the protonated C5-H5 and C6-H6 groups in pyrimidines, C8-H8 groups in purines, and C2-H2 groups in adenosines (Figure 7B).

In the second step, ^15^N-^1^H imino and amino resonances are correlated with the assigned carbons and C-H groups using two different experiments. The 3D (H)CNH experiment (Figure 7C) starts with a long ^1^H-^13^C CP step to transfer proton magnetization to non-protonated carbons. After t_1_ evolution on ^13^C, magnetization is transferred to directly attached amino or imino nitrogens *via* a ^13^C,^15^N CP step. After t_2_ evolution on nitrogens, a phase-cycled selective inversion pulse selects either amino or imino magnetization that is subsequently transferred by a short read-out CP to ^1^H for the detection during t_3_. This experiment yields C6-H6-H61/H62 correlations for A, C4-H4-H41/H42 for C, C2/C6-N1-H1 and C2-N2-H21/H22 for G, and C2/C4-N3-H3 for U (Figure 7D). The correlation of C2/C4 resonances in uridines and C2/C6 resonances in guanosines with imino groups is sufficient for site-specific assignment of imino resonances if chemical shift dispersion exists in at least one of the correlated carbons. Amino groups, on the other hand, have only one adjacent carbon (C6 in A, C4 in C, and C2 in G) with a very narrow chemical shift range, which often prevents unambiguous assignment of amino resonances. This is aggravated by the poor resolution of amino resonances compared to imino resonances. To resolve the ambiguity in these cases, we have combined the two experiments reported above to develop 3D 1) (H)N(C)CH and 2) H(NC)CH experiments. In experiment 1) (Figure 7E), after an initial short ^1^H-^15^N CP step, the chemical shifts of amino and imino nitrogens are recorded during t_1_. Subsequently, magnetization is transferred to directly attached carbons by a ^15^N, ^13^C CP step. The phase-cycled selective inversion pulse selects nucleobase carbon magnetization at ∼ 160 ppm, which is then spread to all nearby carbons by a 14 ms RDFR mixing step. Following this, ^13^C magnetization is evolved during t_2_ and finally transferred from protonated carbons by a short read-out CP to directly attached protons for the detection during t_3_ (Figure 7E). Despite the low sensitivity of this experiment due to the modest efficiency of both low-γ ^13^C,^15^N CP and ^13^C,^13^C RFDR transfers, it provides important information by correlating imino and amino nitrogens directly with the well-resolved C6-H6 and C5-H5 groups in pyrimidines, C8-H8 groups in pyrimidines, and C2-H2 groups in adenosines (Figure 7F). In experiment 2), chemical shifts of amino and imino protons are evolved instead of nitrogens and should provide better spin system separation due to better resolution in the proton dimension. Unfortunately, its sensitivity was poor due to the short coherence lifetimes of imino (∼4 ms) and especially amino (∼1 ms) protons at 100 kHz MAS. Relaxation behavior of RNA amino/imino groups could be improved by 1) increasing the MAS frequency and 2) performing experiments in partial deuterated buffer (∼25–50%), whereas the overall sensitivity of the experiment can be improved by utilizing optimized ^13^C,^13^C recoupling schemes.

While ^13^C,^13^C recoupling-based experiments were successfully utilized for the assignment of most nucleobase resonances, some nuclei can be more efficiently assigned through ^1^H,^1^H recoupling schemes. Thus, amino groups of guanosines were assigned through correlation with well-resolved imino groups through the 3D (H)N(HH)NH experiment described above (Figures 7G,H). Similarly, amino groups of cytidines were assigned through the previously described 3D (H)N(HH)CH experiment (Figure 7I). This experiment provides N4–C5–H5 correlations for all four cytidines in the structured region of 26mer box C/D RNA (Figure 7J) and, moreover, provides assignment of WC A:U (Figures 2G,H) and non-WC G:A base pairs.

In the third step, assignment of non-protonated nitrogen resonances is performed. In ^1^H-detected ssNMR, it can be obtained from a modified version of the 3D (H)NCH experiment described above (Figure 7K). For effective excitation of N1, N3, and N7 resonances, the ^15^N carrier frequency should be shifted to a higher ppm (e.g., ∼190 ppm). Moreover, a selective ^13^C refocusing pulse applied after t_2_ eliminates unwanted signals of the ribose ring. The experiment correlates N7 resonances with protonated C8-H8 groups in purines and N1 and N3 resonances with protonated C2-H2 groups in adenosines. N3 resonances in guanosines, however, do not have any adjacent CH group and can therefore not be assigned in this experiment. The same applies to N3 resonances in cytidines. As in ^13^C-detected ssNMR, assignment of these resonances remains challenging, but N3 resonances of cytidines can likewise be obtained indirectly through base-paired guanosines. Here, the close distance of cytidine N3 and H1 resonances in the base-paired guanosine (r = 2.0 Å) can be exploited in a simple 2D ^1^H-^15^N-HSQC experiment with long-range ^1^H-^15^N/^15^N-^1^H CP transfer times of 8 ms (Aguion et al., 2021).

At intermediate MAS rates, nucleobase assignment can be performed either by acquisition of ^13^C-detected NC-type experiments (Marchanka et al., 2013; Zhao et al., 2019), taking into account attenuated ^13^C sensitivity, or using ^1^H detection of amino/imino protons employing 3D (H)CNH and 3D (H)N(HH)NH experiments. ^1^H-detection of nucleobase H6/H8/H2 protons would be unfavorable due to their short coherence lifetimes at MAS < 60 kHz (Marchanka et al., 2018b).

### Sequential Assignment

**FIGURE 8:**
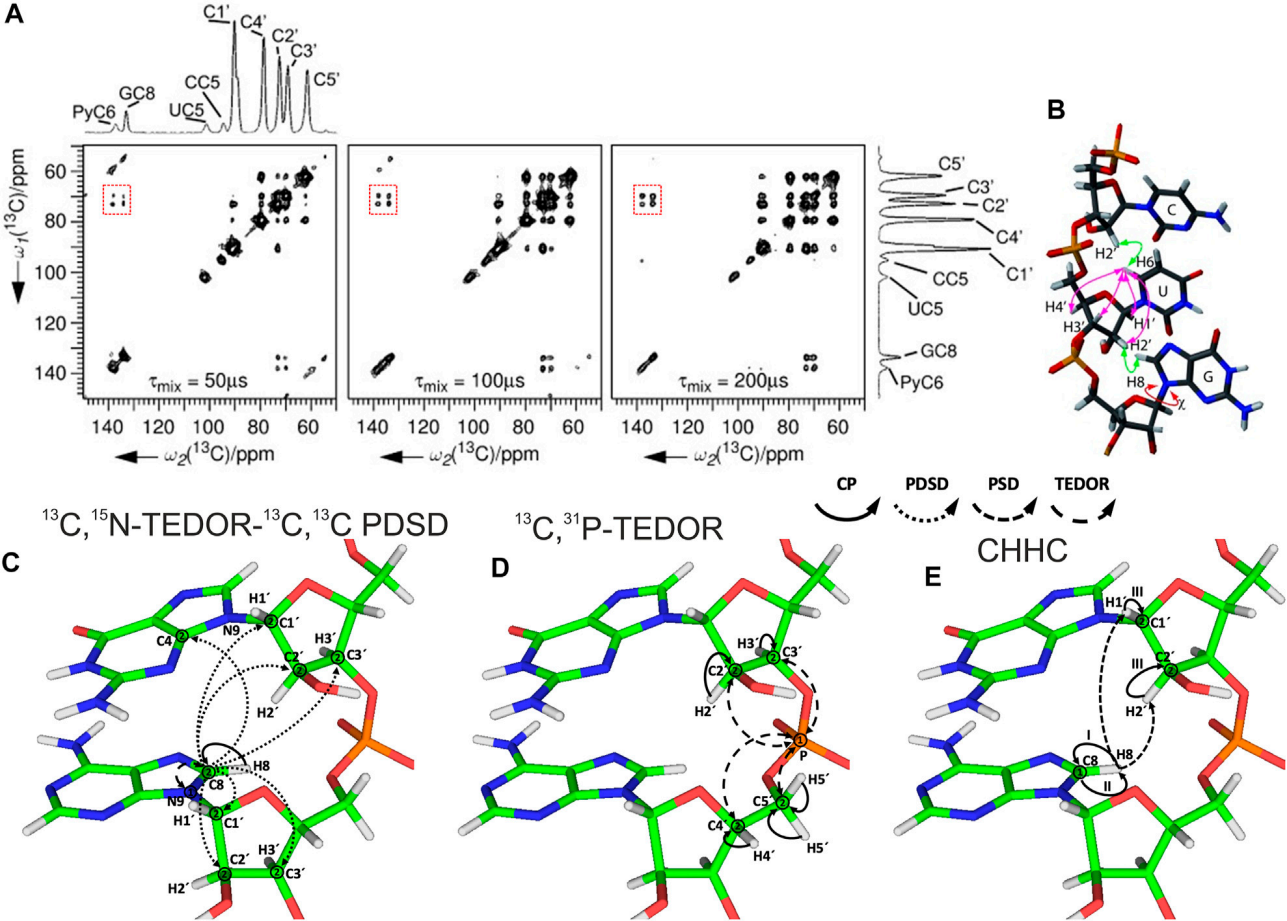
Sequential assignment of RNA by ssNMR. **(A)** CHHC spectra of (CUG)_97_ RNA acquired at different mixing times. The assignments of the different resonances are indicated on the spectral projection shown. Sequential correlations are highlighted. **(B)** Intra- and internucleotide ^1^H–^1^H correlations in a double-stranded A-form helical RNA (Riedel et al., 2006). **(C–E)** Magnetization transfer in **(C)** ^13^C,^15^N-TEDOR-^13^C,^13^C PDSD, **(D)** ^13^C,^31^P TEDOR, and **(E)** CHHC experiments shown on the example of sequential guanosine and adenosine (Marchanka et al., 2015). Solid arrows indicate CP transfers, dotted arrows indicate homonuclear ^13^C,^13^C PDSD transfer, densely dashed arrows indicate PSD transfer, and dashed arrows indicated TEDOR transfer; Arabic numbers correspond to the spectral dimensions (t_1_–t_2_); Roman numbers indicate CP transfers. Panels **(A)** and **(B)** are reprinted with permission from (Riedel et al., 2006) © John Wiley and Sons, 2006.

After spin system–specific assignment is achieved, nucleotides are sequentially connected with each other to obtain site-specific assignments. In solution-state NMR, this task is accomplished by measurement of either through-space internucleotide H6/H8(i)-H2′,H1′(i−1) NOEs or through detection of overlapped C_ribose_–P correlations (Fürtig et al., 2003). A similar approach has been utilized in ssNMR; however, due to the preservation of direct dipolar couplings, accessible distances are significantly longer and are not limited to ^1^H,^1^H restraints. The first ever sequential RNA correlations by ssNMR were obtained by the Görlach group. They have utilized a CHHC-type experiment (Lange et al., 2002) and have measured sequential H2′,H3′(i-1)-H6/H8(i) contacts in the (CUG)_97_ repeat (Figures 8A,B) (Riedel et al., 2006). In our ^13^C- and ^15^N-detected study on 26mer box C/D RNA, we have utilized ^13^C,^13^C and ^13^C,^31^P correlations to obtain sequential assignments (Marchanka et al., 2015). Due to severe resonance overlap, eight different double nucleotide-type selective labeled samples were prepared and an ^15^N-editing TEDOR step was coupled to the long-range ^13^C,^13^C PDSD recoupling (Figure 8C). Long-range correlations up to 9 Å were obtained at 700 ms of PDSD mixing time. While ^13^C,^31^P correlations acquired by the ^13^C,^31^P TEDOR experiment (Figure 8D) provided important sequential contacts in the non-canonical region of RNA (kink-turn) and corroborated sequential assignments obtained by ^13^C,^13^C correlation experiments, their value for correlating nucleotides in the helical regions was limited due to strong resonance overlap. Additional ^1^H,^1^H sequential contacts have been obtained from CHHC and NHHC experiments acquired at 200 us of mixing time (Figure 8E). ^13^C,^31^P and CHHC/NHHC correlation experiments were essential to distinguish sequential contacts from the long-distance inter-strand correlations.

While ^1^H-detected ssNMR allows spin system–specific assignment of both riboses and nucleobases from single, uniformly ^13^C,^15^N labeled RNA samples (Marchanka et al., 2018b; Aguion et al., 2021), no sequence-specific assignment of RNA by ^1^H-detected ssNMR has been reported to date. This objective can be achieved by utilizing, for example, ^1^H,^1^H recoupling coupled with ^13^C-editing either on one (3D) or both sides (4D) to overcome resonance overlap. Furthermore, ^13^C-^31^P and ^1^H-^31^P correlation experiments may be beneficial at fast MAS due to effective averaging of CSA. Our group is currently working in this direction, and we hope to present procedures for the sequential assignment and measurement of structural restraints based on ^1^H-detected ssNMR in the near future.

## Discussion and Outlook

Feasibility of ssNMR approaches for RNA studies largely depends on the quality of sample preparation. While nano/microcrystalline crystallization of rigid RNAs or RNA in RNP complexes (Marchanka et al., 2013) and EtOH precipitation of rigid well-folded RNAs (Zhao et al., 2019) yield well-dispersed spectra, these methods might be less suitable for small RNAs without a well-defined structure, as they provide spectra with narrow chemical shift dispersion (Huang et al., 2012; Yang et al., 2017). This can be an indication of partial loss of the tertiary structure upon PEG or EtOH precipitation, which may limit the advantages of ssNMR methods. Complementary methods of sample preparation, for example, sedimentation of the dissolved sample directly into the ssNMR rotor by ultracentrifugation (Bertini et al., 2011; Wiegand et al., 2020), should be evaluated for their feasibility toward RNA.

Access to narrow RNA resonances makes ssNMR assignment feasible both by ^13^C/^15^N-detected and ^1^H-detected experiments. While conventional ^13^C- and ^15^N-detected studies at MAS < 20 kHz require preparation of many different samples, ^1^H-detected ssNMR at MAS ≥ 100 kHz allows straightforward assignment of small (< 30 nt) RNA using a single uniformly ^13^C,^15^N-labeled sample. We find the intermediate MAS regime of 20 kHz < ω_R_ < 60 kHz suboptimal for RNA studies due to the decreased sensitivity from using ^13^C-detection and the broad ^1^H lines caused by the short coherence lifetime from using ^1^H-detection. Coherence lifetimes of RNA protons increase nearly linearly in the 20–110 kHz MAS range (Marchanka et al., 2018b), and recent protein studies indicate that such a linear regime continues up to at least 150 kHz MAS (Schledorn et al., 2020). ^1^H-detected ssNMR studies at MAS frequencies beyond 110 kHz will further improve spectral quality due to better effective averaging of dipolar ^1^H–^1^H interactions and improved homogeneous linewidth.

Most of the reported ssNMR RNA studies were performed on protonated, nucleotide-type selective or uniformly ^13^C,^15^N labeled RNA. High hydrogen density in the RNA ribose leads to crowded H2′-H5′/H5″ resonances, which are additionally broadened by strong ^1^H–^1^H dipolar couplings. As protein deuteration is beneficial for ^1^H-detected ssNMR studies (Andreas et al., 2015), particularly at MAS < 100 kHz (Cala-De Paepe et al., 2017), selective ribose deuteration as implemented by the Williamson group (Tolbert and Williamson, 1996; Tolbert and Williamson, 1997) can be advantageous for the spectral quality of RNA ribose resonances in ssNMR due to reduction of their spectral overlap and dilution of the ^1^H–^1^H couplings network. Furthermore, sparse labeling of ribose carbons can remove spectral overlap in C2′/C3′ carbons and reduce the dense network of ^13^C,^13^C dipolar- and J-couplings, consequently improving linewidths (Tolbert and Williamson, 1997; Davis et al., 2005) and eliminating dipolar truncation (Bayro et al., 2009). In addition to selective labeling of ribose, specific ^13^C and ^2^H labeling of nucleobases can be beneficial for ssNMR studies. Selective nucleobase deuteration can reduce spectral crowding and improve linewidth of H6 pyrimidine resonances by specific deuteration of H5 protons. Furthermore, atom-specific ^13^C/^15^N labeling of nucleobases, though tedious to synthesize, can remove resonance overlap and facilitate sequential assignment and acquisition of long-range distance restraints (Wunderlich et al., 2012; Juen et al., 2016). Increasing commercial availability of phosphoramidites with different labeling patterns together with the availability of RNA chemical synthesis machinery will allow wide usage of ssNMR RNA studies on atom-specific labeled RNA in the near future.

So far, the presented studies were mostly limited to small RNAs < 30 nt in length with only a few examples of larger RNA, for example, 72 nt long guide RNA in box C/D complex (Marchanka et al., 2018a) or 71 nt long riboA71 (Zhao et al., 2019). While ssNMR can, in principle, be applied to RNA of any size, ssNMR spectra of longer RNA will ultimately have extreme spectral crowding even if nucleotide-type selective labeling is utilized. For such RNAs, nucleotide-type selective labeling should be coupled with segmental labeling (Tzakos et al., 2007; Nelissen et al., 2008; Duss et al., 2010), which will reduce spectral crowding and make complete assignment and structural studies of RNA feasible. This strategy can report on short isotope-labeled RNA stretches exclusively in large RNA or protein–RNA complexes, thereby providing valuable structural information that may be out of reach for other structural biology techniques.
